# Dental and Craniofacial Findings in Early Childhood in Oral–Facial–Digital Syndrome Type 1: A Case Report

**DOI:** 10.1155/crid/8342997

**Published:** 2025-11-28

**Authors:** Selin Saygili, Yelda Kasimoglu

**Affiliations:** ^1^Institute of Graduate Studies in Health Sciences, Istanbul University, Istanbul, Turkey; ^2^Faculty of Dentistry, Department of Pedodontics, Istanbul University, Istanbul, Turkey

**Keywords:** bifid tongue, craniofacial anomalies, delayed tooth eruption, dental arch misalignment, droopy ears, Oral–Facial–Digital Syndrome Type 1

## Abstract

**Background:**

Oral–Facial–Digital Syndrome Type 1 (OFD1) is a genetic disorder marked by diverse malformations of the oral cavity, face, and digits.

**Case:**

This case report presents a female patient who was first referred to the Department of Pedodontics at Istanbul University at 18 months of age due to the absence of teeth in the upper molar region and who has been followed up until 4 years and 11 months. The patient, born prematurely to a consanguineous union, had a history of low birth weight, asphyxia, and a prolonged 2-month intensive care unit stay. Clinical examination revealed multiple dysmorphic features, including facial anomalies such as dolichocephaly, macrocephaly, saddle nose deformity, droopy ears, and thin hair and eyebrows. Intraoral evaluation demonstrated misalignment of the dental arches, abnormal tooth positioning, a bifid lobulated tongue, and residual sutures from previous cleft palate surgery. Although the upper primary central incisors were present, the lower incisors were initially absent, with subsequent follow-ups showing their delayed eruption. At 39 months, further evaluation revealed malposed and rotated upper primary dentition, while certain lower deciduous central incisors remained undetectable. The patient was managed with protective dental care, regular 3-month follow-ups, and comprehensive parental education on oral hygiene, alongside coordinated consultations with orthodontic services.

**Conclusion:**

This report highlights the clinical heterogeneity of OFD1 and underscores the importance of early diagnosis, multidisciplinary management, and continuous monitoring to improve oral health outcomes in affected patients.

## 1. Introduction

Oral–facial–digital syndrome represents a diverse array of conditions affecting the mouth, face, and digits. To date, researchers have identified at least 16 distinct subtypes, which vary based on their inheritance patterns and are often associated with abnormalities in the kidneys, limbs, brain, and other organs (Table 1) [2]. The most prevalent subtype, Oral–Facial–Digital Syndrome Type 1 (OFD1) (Online Mendelian Inheritance in Man [OMIM] ID #311200), is primarily inherited through an X-linked dominant pattern [3, 4]. OFD1 is a genetic disorder affecting development, which was initially identified by Papillon–Leage and Psaume in 1954 [5] and later detailed by Gorlin and Psaume in 1962 [6]. Genetic heterogeneity exists in female patients with phenotypic variation due to X-inactivation, but it is lethal in embryonic males [7]. Notably, over 70% of OFD1 cases occur sporadically [8]. OFD1 is estimated to occur in about one out of every 50,000 live births [9].

OFD1 is uniquely characterized by features such as congenital milia and hypotrichosis, which are not found in other subtypes [10]. Additionally, this condition commonly manifests in females and is distinguished by abnormalities in the mouth, face, and digits [3].

OFD1 is distinguished by a range of characteristics including oral anomalies (such as a lobulated tongue, nodules on the tongue, clefts in the hard or soft palate, extra gingival frenulae, missing teeth, and other dental irregularities); facial attributes (telecanthus, underdeveloped alae nasi, a central cleft or pseudocleft of the upper lip, and a small lower jaw); digital abnormalities (short fingers, fused fingers, bent fifth finger, and a duplicated big toe); polycystic kidney disease; brain MRI results showing brain cysts, absence of the corpus callosum, absence of the cerebellum with or without Dandy–Walker malformation; and intellectual disability, which occurs in about half of the individuals with this condition [11].

## 2. Case Report

### 2.1. Patient History

An 18-month-old female patient was referred to the Department of Pedodontics, Faculty of Dentistry, Istanbul University, due to the absence of teeth in the upper molar region. The patient is the fifth child of a potentially consanguineous union and was born to a 25-year-old mother, with congenital contractural arachnodactyly detected during the antenatal period at 32 weeks of gestation. The patient was born prematurely, had a low birth weight, and required a 2-month stay in the intensive care unit. Corpus callosum agenesis and developmental delay are present, although no additional systemic diseases have been identified. Genetic analysis revealed a heterozygous mutation (13c1411+16>A).

Her history revealed mild developmental retardation, with independent standing and walking achieved at 15 months. At 17 months, she underwent first cleft palate repair surgery. In a subsequent appointment, her mother reported that the patient was undergoing speech and language therapy.

### 2.2. Extraoral Findings

**Figure 1:**
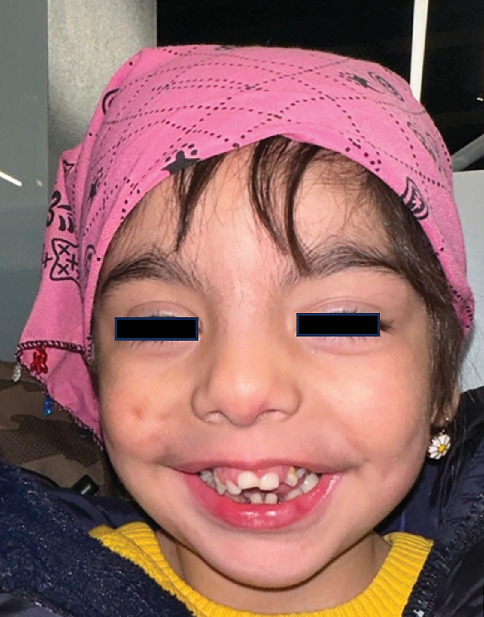
Frontal facial image showing open bite and small mandible. A frontal view of the patient's face, demonstrating a visibly smaller lower jaw and open bite malocclusion, features that align with the craniofacial characteristics of Oral–Facial–Digital Syndrome Type 1.

**Figure 2:**
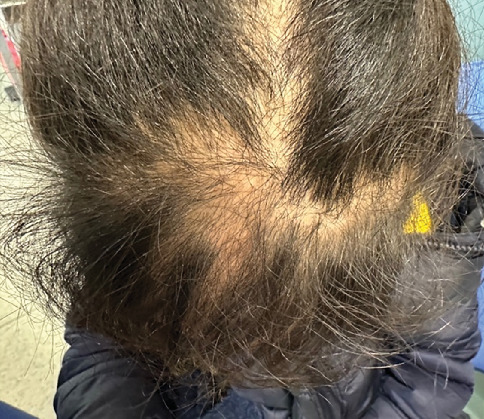
Overhead view demonstrating sparse hair in a patient with OFD1. A top–down photograph revealing thinning and patchy hair, illustrating hypotrichosis—a feature frequently associated with Oral–Facial–Digital Syndrome Type 1.

Physical examination demonstrated several dysmorphic features, including facial anomalies (Figure 1), dolichocephaly, macrocephaly, saddle nose deformity, droopy ears, and thin eyebrows and sparse hair (Figure 2). Parental consent was obtained for the publication of all photographic materials.

### 2.3. Intraoral and Radiographic Findings

**Figure 3:**
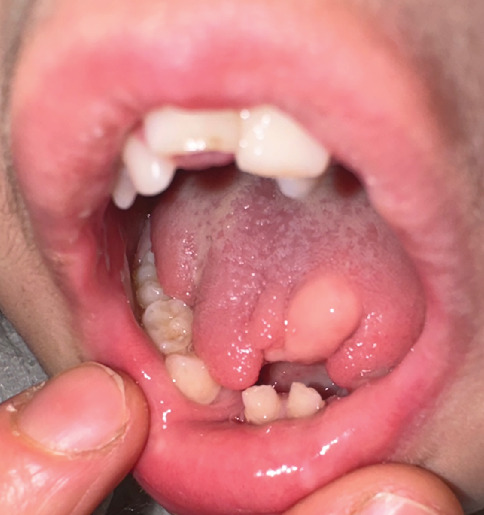
Bifid, lobulated tongue at 18 months with hamartomatous nodule. Intraoral photograph highlighting the bifid lobulated tongue and a pinkish nodule on the lateral surface, consistent with a hamartomatous lesion. This presentation underscores the oral abnormalities associated with the patient's condition at this early age.

**Figure 4:**
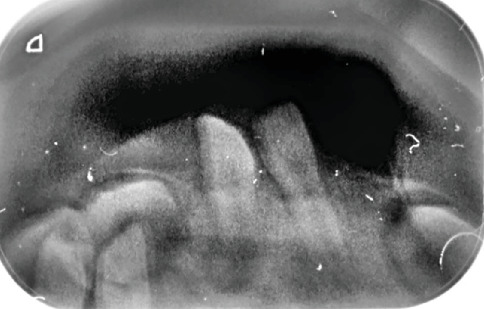
Initial periapical radiograph revealing developing lower incisors. A periapical radiograph taken at the first visit, demonstrating the presence of the lower incisors beneath the gum line despite their clinical absence, thereby confirming ongoing dental development at this early stage.

During the initial intraoral examination, misalignment of the dental arches, abnormal tooth positioning, a bifid lobulated tongue, normochromic to slightly pinkish nodule on the lateral surface of the tongue, and evidence of previous cleft palate surgery were noted (Figure 3). Periapical radiography was performed at this appointment (Figure 4). Although the upper primary central incisors were present, the lower incisors had not yet erupted. A later panoramic radiograph obtained at 4 years and 11 months demonstrated deep dentinal caries in six teeth (LRE, LRD, LLD, LLE, URE, and ULE) and possible germ absence of the teeth lower left second premolar (LL5) and lower right second premolar (LR5).

### 2.4. Treatment and Follow-Up

**Figure 5:**
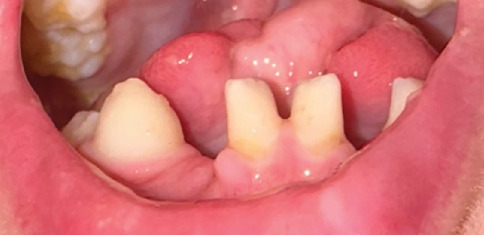
Lower incisor eruption at follow-up. Intraoral photograph taken at a subsequent appointment, illustrating the previously absent lower incisors now emerging in the mandibular arch. This confirms delayed but ongoing tooth development and highlights the need for continued monitoring of tooth eruption patterns.

**Figure 6:**
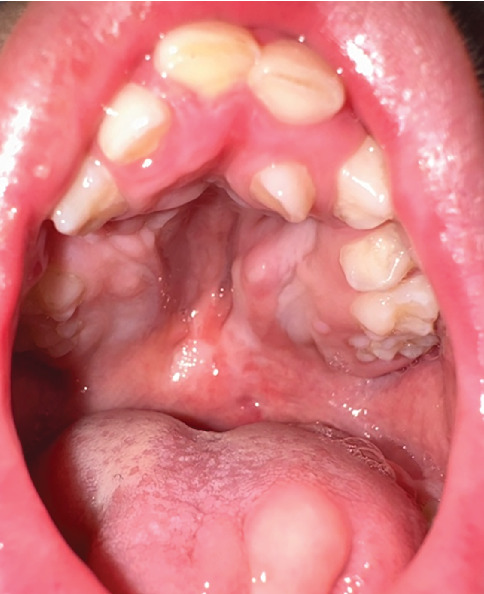
Clinical photograph at 39 months illustrating malposed upper primary dentition and deep V-shaped palate.

In subsequent follow-ups, the eruption of the lower incisors was observed (Figure 5). At the 39-month appointment, clinical evaluation revealed that the upper primary dentition had erupted in a malposed and rotated manner, while teeth lower left deciduous central incisor (LLA) and lower right deciduous central incisor (LRA) were not visible in the lower arch. Additionally, a later intraoral examination demonstrated a deep V-shaped palate, clearly separated from the anterior segment, along with ankyloglossia, irregular morphology of the primary teeth, and tooth rotations (Figure 6).

The patient received protective dental care. Both the patient and her parents were informed about the significance of ongoing oral hygiene and regular dental visits to prevent dental issues. A treatment plan was devised for the patient based on thorough evaluation, and the parents were briefed about it. Additionally, we coordinated with other relevant departments, such as orthodontics, considering the patient's specific dental condition.

In addition to dental and orthodontic follow-up, the patient was also monitored by pediatric neurology due to corpus callosum agenesis and developmental delay and received ongoing speech and language therapy following cleft palate repair. Genetic consultation was provided to the family. Routine systemic evaluations were performed to screen for other organ involvement associated with OFD1, such as renal anomalies, but no additional findings were detected during follow-up.

**Figure 7:**
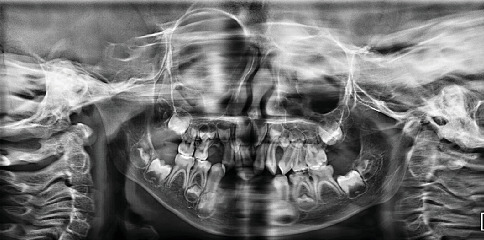
Panoramic radiograph at 4 years 11 months. The image demonstrates dentition with multiple deep dentinal caries. Atraumatic restorative treatment was performed on affected teeth. Due to the patient's limited cooperation, the radiograph quality is suboptimal, yet diagnostic information could still be obtained. Possible germ absence of Teeth LL5 and LR5 was suspected at this stage.

At the follow-up visit at the age of 4 years and 11 months, panoramic radiography was obtained (Figure 7). Deep dentinal caries were detected in multiple teeth. Considering the patient's age and limited cooperation, atraumatic restorative treatment was performed chairside to manage the lesions in a minimally invasive manner. Given the patient's young age and caries risk, we will continue 3-monthly reviews focused on prevention and behavior guidance. Topical fluoride varnish will be applied routinely at each recall. For cavitated lesions in noncooperative sessions or where definitive treatment must be deferred, we will consider silver diamine fluoride (SDF) to arrest progression. As cooperation improves and with the eruption of the permanent first molars, we plan early fissure sealants. We also arranged orthodontic consultation to monitor arch form, rotations, and potential effects of the cleft and ankyloglossia on occlusal development. The patient will remain under shared care (pediatric neurology, speech therapy, and genetics), with annual panoramic or periapical reassessment as tolerated to clarify the presence or absence of premolar germs (LL5/LR5).

## 3. Discussion

### 3.1. Clinical Manifestations in OFD1 Patients

Various forms of this syndrome have been documented in the literature, yet its complete spectrum remains unclear. Type I is the most frequently observed variant, with autosomal recessive inheritance being the most common among its subtypes [12].

The current case exhibited multiple clinical manifestations, with particularly pronounced oral features alongside some facial characteristics. It is important to note that certain facial features may be absent or present with varying intensities in other cases. Notably, digital abnormalities were absent in our patient. Ko et al. [1] described an 11-month-old female presenting with multiple milia on her face and auricle from birth. Although our patient's primary concerns centered on dental anomalies, similar skin findings may be part of the overall clinical presentation.

### 3.2. Oral Hamartomas in OFD

Hamartomas are benign growths made up of fully developed tissues normally found in that area. For example, although the tongue usually contains only a few isolated fat cells, hamartomatous lesions have a high concentration of adipocytes that blend with the surrounding normal tissue [13].

In the mouth, hamartomas are uncommon, but the tongue—particularly its upper surface—is the most frequent site in children. They typically show up as painless, skin-colored nodules with a smooth appearance, though tongue hamartomas can also appear as exophytic, nodular, or polypoid formations, and may be either broadly attached or on a stalk [13]. Within the broad spectrum of clinical presentations observed in these conditions, tongue hamartomas have been identified in Types I through IX [14]. Additionally, tongue hamartomas have been linked to ectrodactyly-ectodermal dysplasia-cleft syndrome [15] and have been observed in patients with craniofacial clefts and bifid tongues [16, 17].

In our case, considering the young age of the patient and the absence of any clinical complications associated with the lesion, it was decided that surgical excision in collaboration with the Oral and Maxillofacial Surgery Department was not necessary at this stage.

### 3.3. Management and Treatment Considerations

Managing patients with OFD presents significant challenges. When the disease-causing mutation is identified within a family, prenatal diagnosis becomes feasible for at-risk pregnancies, and prenatal ultrasound may reveal structural brain anomalies or hallux duplication. Depending on the clinical presentation, treatment may involve cosmetic or reconstructive surgery for lip and/or palate clefts, excision of tongue nodules, and removal of accessory frenulae; management similar to that for isolated cleft palate—including speech therapy and aggressive treatment of otitis media—may also be indicated. Orthodontic intervention for malocclusion and corrective surgery for syndactyly, if present, might be necessary. In cases where renal disease develops, interventions such as hemodialysis, peritoneal dialysis, or renal transplantation may be required. OFD1 is a multisystem disorder that frequently includes cutaneous manifestations.

In our patient, multidisciplinary care involved pediatric neurology, speech and language therapy, and genetic counseling, in addition to dental management. Although renal involvement is well recognized in OFD1, no abnormalities were identified during follow-up. This emphasizes the need for coordinated medical and dental surveillance to address both syndrome-related anomalies and routine oral health problems.

Follow-up at 4 years and 11 months revealed multiple deep dentinal caries, which were managed with atraumatic restorative treatment. This highlights that, in addition to the congenital anomalies inherent to OFD1, patients may also be prone to an increased risk of dental caries due to difficulties in maintaining oral hygiene and cooperation challenges during treatment. Obtaining radiographs was particularly difficult in this case because of limited cooperation, which resulted in images of suboptimal quality. Minimally invasive techniques such as atraumatic restorative treatment can therefore play a valuable role in stabilizing the oral condition and preserving function, while comprehensive preventive strategies and regular monitoring remain essential.

## 4. Conclusion

This case underlines the critical role of pediatric dental professionals in the early detection and long-term management of dental anomalies in OFD1. Moreover, a multidisciplinary approach involving orthodontists, speech therapists, and other relevant specialists is essential for addressing the complex needs of these patients. Continuous follow-up and a tailored treatment strategy are vital to meet both the immediate and future requirements, ensuring improved oral health and quality of life.

## Figures and Tables

**Table 1:** Sixteen subtypes of oral–facial–digital syndrome. Overview of the 16 recognized subtypes of oral–facial–digital syndrome, detailing their inheritance patterns, chromosomal locations, associated genes, and characteristic clinical findings. Adopted from Ko et al. [1].

**Phenotype**	**Inheritance**	**Location**	**Gene/locus**	**Characteristic clinical finding**
OFD1	X-linked dominant	Xp22.2	OFD1, SGBS2, JBTS10, RP23	Milia, hypotrichosis, polycystic kidney disease
OFD2	Autosomal recessive	Not mapped	OFD2	Thick hair, median Y-shaped metacarpal
OFD3	Autosomal recessive	Not mapped	OFD3	End-stage renal failure I-II decade of life
OFD4	Autosomal recessive	10q24.1	TCTN3, TECT3, C10orf61, OFD4, JBTS18	Renal cyst
OFD5	Autosomal recessive	1q32.1	DDX59, OFD5	
OFD6	Autosomal recessive	5p13.2	CPLANE1, C5orf42, JBTS17, OFD6	Broad hallux, median Y-shaped metacarpal
OFD7	X-linked dominant	Not mapped	OFD7	Polycystic kidney disease
OFD8	X-linked recessive	Chromosome X	OFD8	Tibia and radius hypoplasia
OFD9	Autosomal recessive	Not mapped	OFD9	Bifid toes, microphthalmia, coloboma
OFD10	Autosomal dominant	Not mapped	OFD10	Bilateral short radius, fibular agenesis
OFD11	Isolated cases	Not mapped	OFD11	Odontoid hypoplasia, deafness
OFD14	Autosomal recessive	11q13.4	C2CD3, OFD14	
OFD15	Autosomal recessive	17p13.1	KIAA0753, OFIP, OFD15	
OFD16	Autosomal recessive	17p13.1	TMEM107, MKS13, JBTS29	
OFD17	Autosomal recessive	4q28.1	INTU, KIAA1284, PDZK6, SRTD20, OFD17	
OFD18	Autosomal recessive	3q13.12-q13.13	IFT57, ESRRBL1, HIPPI, OFD18	

## Data Availability

Data sharing is not applicable to this article as no datasets were generated or analyzed during the current study.

## Nomenclature

OFD1: Oral–Facial–Digital Syndrome Type 1
OMIM: Online Mendelian Inheritance in Man
